# Annotations

**Published:** 1903-10-24

**Authors:** 


### ANNOTATIONS.

### Massage by the Blind.

We have before now had occasion to call attention to the Japanese custom of employing the blind as masseurs as one worthy of imitation in this country, and we are pleased to notice that an Institute for Massage by the Blind is in process of organisation at the present moment under the patronage of H.R.H. the Duke of Cambridge. It is a fact that blind people become particularly skilful in the performance of massage, partly, no doubt, on account of the well-known circumstance that the loss of one sense usually leads to a special cultivation of the senses that remain. And as massage is an art, the successful performance of which depends in a high degree upon delicacy of touch and manipulative ability, it is one which the blind are pre-eminently suited to perform. The advantages which may be expected from the organisation mentioned are manifold. In the first place, the afflicted individual who has lost the power of vision will be enabled to earn a livelihood and will be provided with a regular occupation and object for existence, without which any degree of happiness is seldom to be found, and, secondly, the community as a whole will benefit by the employment in useful labour of those whose powers would otherwise run to waste. It is intended by the committee of the Institute to secure central rooms which shall be used (1) for the treatment of patients; (2) as a central office from whence operators can be sent out to patients requiring treatment at their own homes, to hospitals and other institutions. In the meantime a sum of about £600 is needed, in order to start the Institute on a proper footing, and it is hoped that ultimately it will be self-supporting. We feel sure that such a worthy institution as this will not have to wait long before the required sum is forthcoming. Contributions should be sent to the Treasurer, F. O. Smithers, Esq., 171 Adelaide Road, South Hampstead, N.W.

### "Electrical" Quacks.

The recent successful prosecution of a person who had once been a member of the medical profession, who had been removed from the register in consequence of his conviction for felony, and who, on his release, entered the service of a dealer in so-called "electrical" apparatus and was advertised in connection with the business as a legally qualified medical man, is an event calculated to give rise to much consideration. It appeared that the accused was employed by some "company" having its domicile abroad, and that his business was to recommend and sell certain widely advertised "electrical" belts, which were offered to the public as cures for many diseases. The cost of making them may have been, perhaps, two shillings or half-a-crown each, and, according to counsel, they were absolutely worthless and inoperative; but the price charged for a "belt" was said to range from £10 to £3, according to the apparent position and the apparent credulousness of the purchaser. Now it is notorious that these things have been very largely advertised in several cheap newspapers, the advertisements being usually pictorial, and representing imaginary lines of force proceeding from the belts in question. We wonder whether it would be quite useless to appeal to the proprietors of these newspapers to neglect the Vespasianic maxim, and no longer to make themselves accomplices in one of the grossest and most barefaced frauds of modern times. Surely our contemporaries may be persuaded to make some small pecuniary sacrifice for the sake of honesty. They may fairly be asked to remember that the chief victims of the advertisements referred to are the poor; and that hundreds of poor people have deprived themselves and their families of necessaries in order to purchase worthless things recommended in the newspaper in which they have come to trust. Such people do not discriminate accurately between the advertisement columns and the editorial portion. To them the "paper" is enough, and they fall victims of the traps laid for them by some of the very basest and vilest of the human race. Such would assuredly not be an unfair description of people who rob the poor and the suffering by holding forth promises of relief which those who make them know can never be fulfilled; and the contemporaries to whom we appeal might easily satisfy themselves of the true character of some of the things that are advertised. It is manifest that the sale of a two shilling article for ten pounds is not likely to be stopped by any fines which can be imposed for the false assumption of a medical character. A prosecution of the advertisers for obtaining money under false pretences might possibly be effectual, were it not that difficulties arising from the foreign domicile would be likely to stand in the way.

### New Complexions for Old.

Yet one more example of the ridiculous lengths to which vanity and weakness may be tempted by the blandishments of the quack appeared recently in the columns of a daily paper, in the form of an article purporting to recount the sufferings and other experiences endured by a lady who, not being satisfied with the beauty of her complexion, had undergone "the wonderful operation of removing the skin of the face." However, it appears that an altogether unaccountable attack of modesty on the part of the operator induced her at the last moment to rest satisfied with merely blistering off the epidermis and leaving the skin behind. The method employed was as follows: The face was first painted with cocaine to deaden the smart (a procedure which of itself displays the ignorance of the operator), and then smeared over with blistering fluid and covered with strips of plaster. As one might have imagined, the result of this so-called beautifying process was, we are told, painful in the extreme, the face swelling to twice its natural size and the patient having to take her tea from a feeding cup, as her mouth "seemed to have disappeared." We fancy that no one in the possession of her mental faculties would be likely, at any rate after reading the article referred to, to present herself as a candidate for such an operation. But vanity is a strange leader and therefore it may be as well to point out, for the benefit of those who are disposed to be discontented with their own natural charms, that the operation of having the face blistered all over is attended with grave dangers of erysipelas and other infections, and, moreover, that the usual effect of blistering is to leave the skin more pigmented than it was before.

